# Supplementary material for: Evaluation of a bone filler scaffold for local antibiotic delivery to prevent Staphylococcus aureus infection in a contaminated bone defect
Source: Sci Rep. 2021 May 13;11:10254. doi: 10.1038/s41598-021-89830-z (PMC8119729; doi:10.1038/s41598-021-89830-z)
Supplement: Supplementary file 1 — Supplementary Information. [file 41598_2021_89830_MOESM1_ESM.pdf]

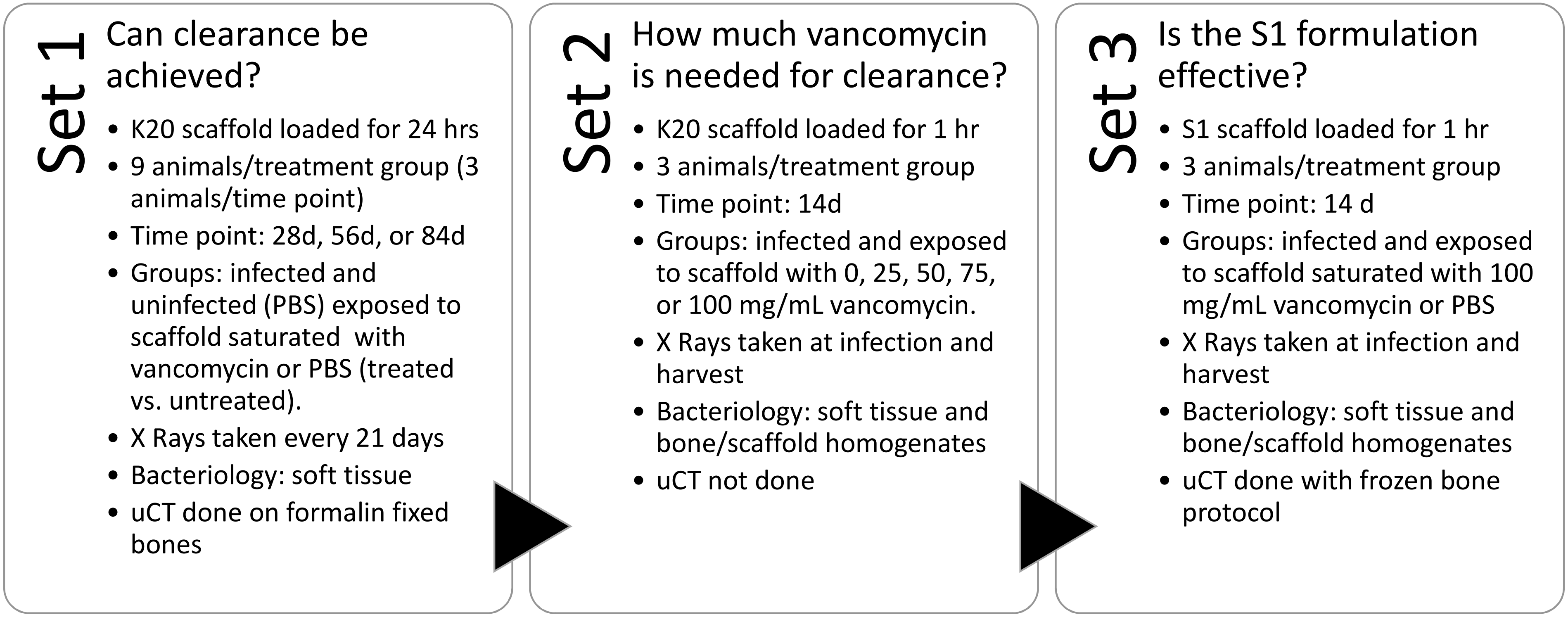

Supplementary Figure S1. Animal Experiment Progression Schematic. The animal experiments were performed in three sets, each aimed at a different question. Improvements to the protocol and differences between the sets are noted.

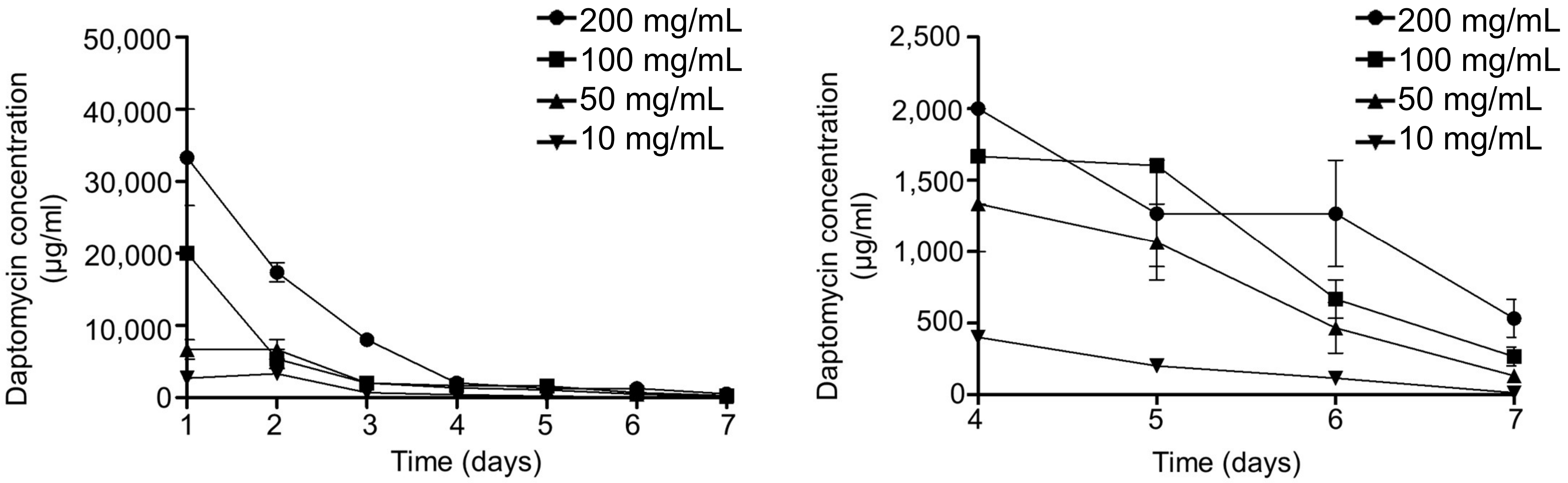

Supplementary Figure S2. Uptake and elution of daptomycin as a function of antibiotic concentration. The K20 scaffold was exposed to solutions of daptomycin at the indicated concentrations for 24 hours. Scaffolds were then removed and placed in PBS without antibiotic. Scaffolds were then removed and placed in PBS without daptomycin. The buffer was replaced with fresh antibiotic-free buffer at the indicated time points and the approximate amount of active daptomycin determined using a broth microdilution bioassay. Left: the results from a 7-day elution period. Right: Exploded view of the data focusing only on the later time points.

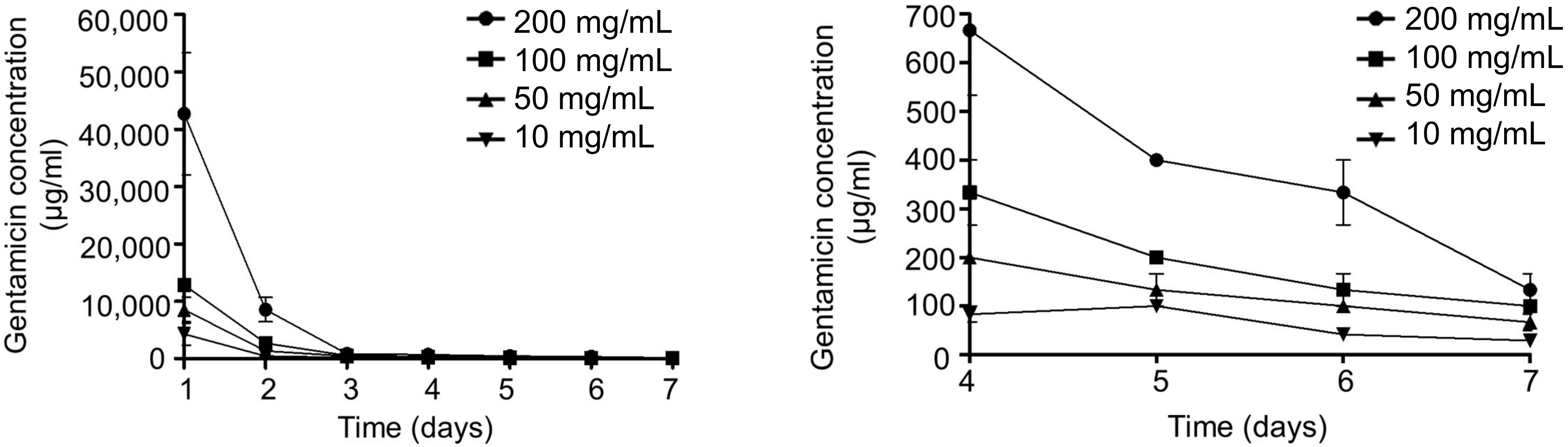

Supplementary Figure S3. Uptake and elution of gentamicin as a function of antibiotic concentration. The K20 scaffold was exposed to solutions of gentamicin at the indicated concentrations for 24 hours. Scaffolds were then removed and placed in PBS without antibiotic. Scaffolds were then removed and placed in PBS without gentamicin. The buffer was replaced with fresh antibiotic-free buffer at the indicated time points and the approximate amount of active gentamicin determined using a broth microdilution bioassay. Left: the results from a 7-day elution period. Right: exploded view of the data focusing only on the later time points.

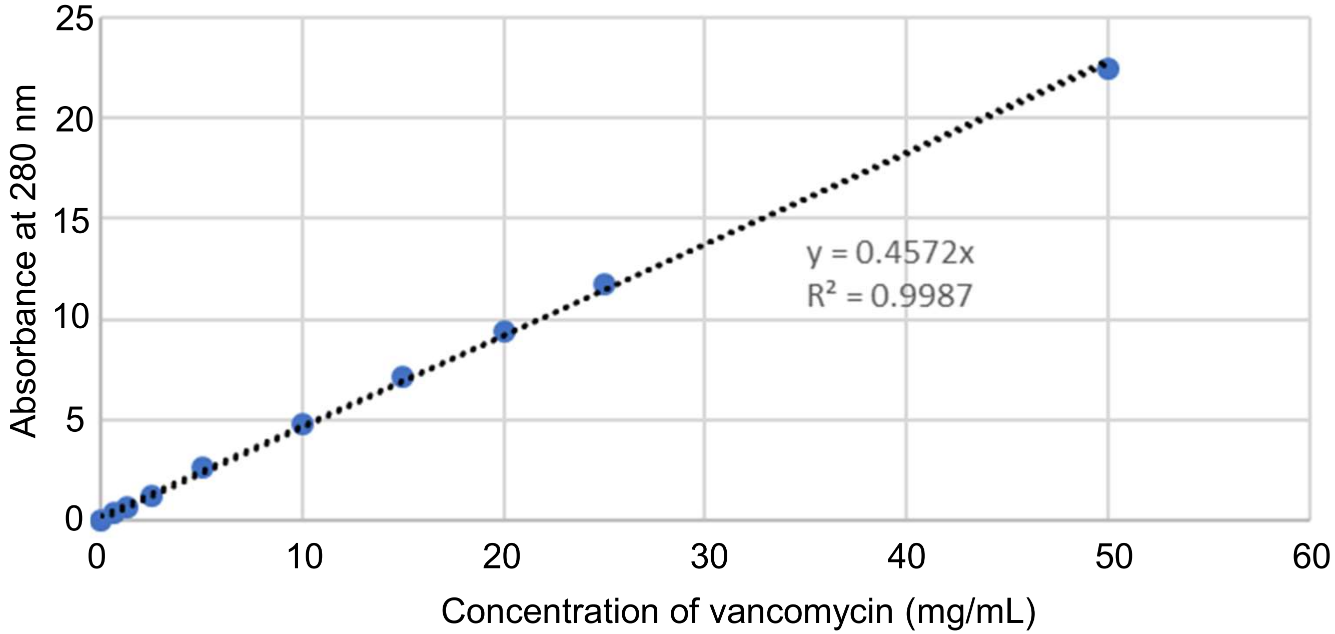

Supplementary Figure S4. Correlation between concentration of active vancomycin absorbance at 280 nm. A solution of vancomycin was freshly prepared in PBS at the indicated concentrations and the absorbance at 280 nm measuring as a function of antibiotic concentration.

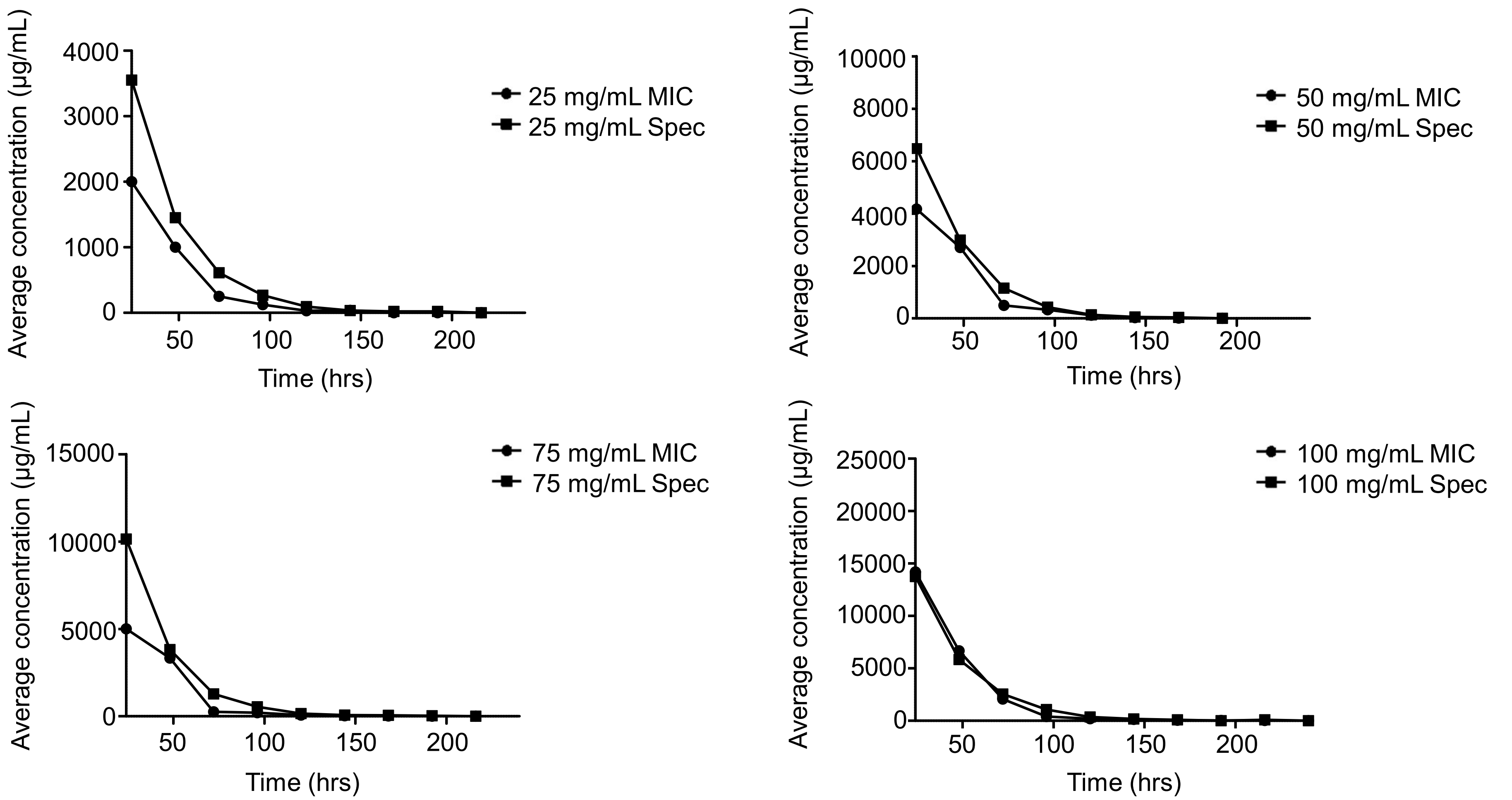

Supplementary Figure S5. Correlation between bioassay and spectrophotometric assay for vancomycin. The K20 scaffold formulation was saturated in a solution containing the indicated amounts of vancomycin for 1 hour at room temperature. Scaffolds were then removed and placed in PBS without vancomycin. The buffer was replaced with fresh antibiotic-free buffer at the indicated time points and the approximate amount of active vancomycin at each sampling point determined by bioassay (MIC) and spectrophotometric analysis (Spec).
